# Serum metabolites reflecting gut microbiome alpha diversity predict type 2 diabetes

**DOI:** 10.1080/19490976.2020.1778261

**Published:** 2020-06-24

**Authors:** Cristina Menni, Jialing Zhu, Caroline I Le Roy, Olatz Mompeo, Kristin Young, Casey M. Rebholz, Elizabeth Selvin, Kari E. North, Robert P Mohney, Jordana T Bell, Eric Boerwinkle, Tim D Spector, Massimo Mangino, Bing Yu, Ana M Valdes

**Affiliations:** a Department of Twin Research and Genetic Epidemiology, Kings College London, London, UK; b School of Public Health, University of Texas Health Science Center, Houston, TX, USA; c Department of Epidemiology, Gillings School of Global Public Health, University of North Carolina, Chapel Hill, NC, USA; d Department of Epidemiology, Johns Hopkins Bloomberg School of Public Health, Baltimore, MD, USA; e Metabolon Inc., Morrisville, NC, USA; f Baylor College of Medicine, Houston, TX, USA; g School of Medicine, Nottingham, UK; h NIHR Nottingham Biomedical Research Centre, Nottingham, UK

**Keywords:** Microbial metabolites, microbiome diversity, prevalent diabetes, incident diabetes

## Abstract

Type 2 diabetes (T2D) is associated with reduced gut microbiome diversity, although the cause is unclear. Metabolites generated by gut microbes also appear to be causative factors in T2D. We therefore searched for serum metabolites predictive of gut microbiome diversity in 1018 females from TwinsUK with concurrent metabolomic profiling and microbiome composition. We generated a Microbial Metabolites Diversity (MMD) score of six circulating metabolites that explained over 18% of the variance in microbiome alpha diversity. Moreover, the MMD score was associated with a significantly lower odds of prevalent (OR[95%CI] = 0.22[0.07;0.70], P = .01) and incident T2D (HR[95%CI] = 0.31[0.11,0.90], P = .03). We replicated our results in 1522 individuals from the ARIC study (prevalent T2D: OR[95%CI] = 0.79[0.64,0.96], P = .02, incident T2D: HR[95%CI] = 0.87[0.79,0.95], P = .003). The MMD score mediated 28%[15%,94%] of the total effect of gut microbiome on T2D after adjusting for confounders. Metabolites predicting higher microbiome diversity included 3-phenylpropionate(hydrocinnamate), indolepropionate, cinnamoylglycine and 5-alpha-pregnan-3beta,20 alpha-diol monosulfate(2) of which indolepropionate and phenylpropionate have already been linked to lower incidence of T2D. Metabolites correlating with lower microbial diversity included glutarate and imidazole propionate, of which the latter has been implicated in insulin resistance. Our results suggest that the effect of gut microbiome diversity on T2D is largely mediated by microbial metabolites, which might be modifiable by diet.

## Introduction

Type 2 diabetes (T2D) is a major public health burden with a 10-fold increase in T2D rates globally between 1980 (30 million) and 2010 (300 million).^1^ Accumulating evidence indicates that bacteria in the human gut play a vital role in the pathophysiology of T2D and related traits,^2–7^ with higher gut microbiome alpha diversity having a beneficial effect.

Gut microbiome composition is regulated jointly by the host genome, the colonic milieu and diet.^6^ During digestion, gut microbes coproduce a wide range of metabolites such as short chain fatty acids (SCFAs),^8^ lipopolysaccharide (LPS),^9^ imidazole propionate,^10^ among others, that are essential for regulating multiple host-microbiome pathways.^11^ Previous studies in animals and humans have identified a gut microbiome signature of T2D.^6,7^ Individuals with T2D have lower gut microbiome alpha diversity compared with healthy controls and they also show impaired SCFA butyrate production,^12^ modifications in incretin secretion, reducing intestinal permeability leading to metabolic endotoxemia (increased LPS in blood)^13,14^ that is linked to increasing metabolic inflammation and insulin resistance induced by high-fat diet.^15^

In addition, a number of studies have shown that microbial metabolites, other than SCFAs, are linked to the development of T2D,^16^ suggesting that part of the benefit of having a diverse gut microbiome is related to an improvement of the metabolic cross-talk occurring between the gut microbes and their host.^17^

A recent study has found that 40 metabolites, including 13 of them of microbial origin^18^ can predict 45% of the inter-individual variation in alpha diversity. However, the study did not focus on the specific relevance of bacterial metabolites to T2D, but primarily on the usefulness of human and bacterial metabolites as biomarkers predictive of diversity.

Here we identify serum metabolites reflective of gut microbiome function that can mediate the effect of gut microbiome composition on T2D and related metabolic traits in a large UK cohort. We replicate our results in an independent US sample.

## Results

The descriptive characteristics of the study populations are presented in Table 1. We included 1018 middle-age females from TwinsUK with 16 s gut microbiome data and concurrent fasting serum metabolomics. The replication cohort consisted of 1522 male and female participants from the ARIC study of European ancestry.

**Table 1.** Descriptive characteristics of the study population.

	TwinsUK	ARIC
	*N (%)*	*N (%)*
N	1018	1522
Females, %	1018 (100%)	827 (54.3%)
T2D prevalent cases,	47 (4.72%)	119 (7.8%)
T2D incident cases	23	467 (34.7%)
	*Mean (SD)*	*Mean (SD)*
Age, yrs	65.19 (7.74)	54.56 (5.74)
BMI, kg/m^2^	26.19 (4.83)	27.25 (5.01)
Homa2-IR	0.93 (0.67)	
Glucose, mmlol/l	4.75 (0.63)	5.84 (1.54)
VFM, (g)	606.77 (290.07)	
*Indices of microbiome diversity*		
Shannon Diversity	5.14 (0.7)	NA
Observed number of OTUs	89.38 (113.52)	NA
Simpson diversity	0.92 (0.06)	NA

**Figure 1.**
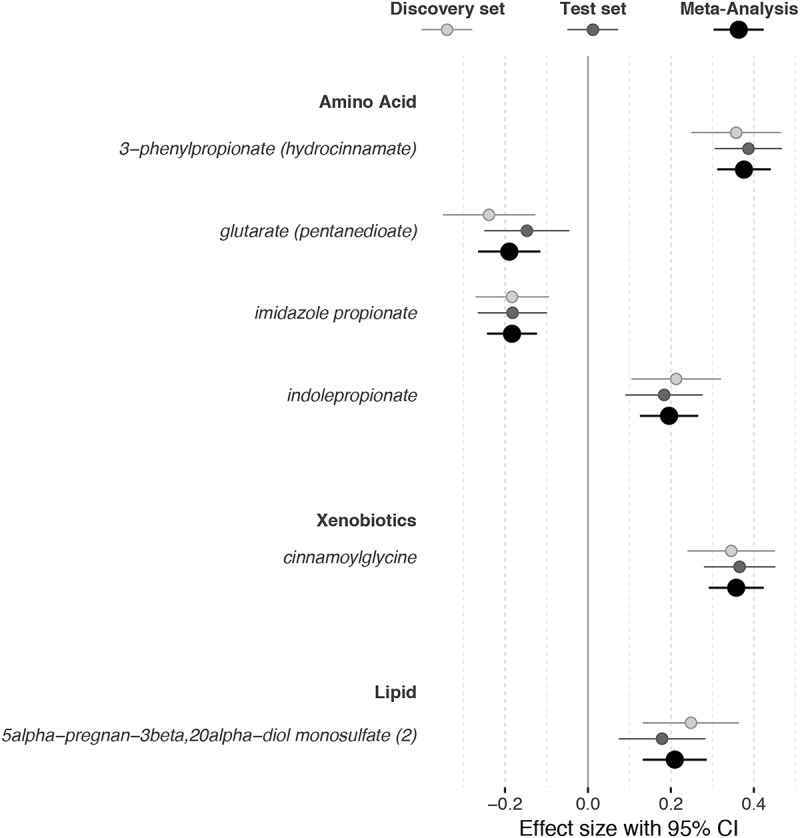
Metabolites trait significantly associated with gut microbiome alpha diversity (Shannon Index) in the discovery and test set of the TwinsUK cohort and in the overall cohort (using inverse variance fixed effect meta-analysis). Analyses adjusted by age, sex, body mass index, family relatedness, and multiple testing. CI indicates confidence interval.

We randomly divided the TwinsUK study sample into two independent sets: a discovery set of 409 individuals and a test set of 609 individuals. Six metabolites were associated with Shannon diversity after adjusting for age, BMI, family relatedness (as random effect) and multiple testing (using Bonferroni correction *P* < 1.68x10^−4^ = 0.1/596 metabolites) in the discovery set (Table S1). Of these, there were four amino acid derivatives, one steroid and one xenobiotic. We then created a microbial metabolite diversity (MMD) score in females from TwinsUK to assess the combined effects of all metabolic traits identified, by including the six metabolites into a linear regression. We found that the MMD score explains 27% of the variance in Shannon Diversity (Figure 1) and the variance inflation factor (VIF) between the selected feature was low (VIF = 1.38) suggesting low collinearity.^19^

$$\begin{array}{rl} & MMD\;score\;=\;-0.0476306+0.1746576\\ & \times \left(3-phenylpropionate\left(hydrocinnamate\right)\right))\\ & -0.0867652\times \left(imidazolepropionate\right)\\ & +\;0.1461903\times \left(cinnamoylglycine\right))\\ & +0.1674953\times \left(\begin{array}{c} 5alphapregnan\_3beta,\\ 20alpha\_diolmonosulfate\left(2\right) \end{array}\right)\\ & -0.145269\times \left(glutarate\left(pentanedioate\right)\right)\\ & +0.0683178\times (indolepropionate) \end{array}$$

We then replicated the Shannon-metabolite association in 609 independent individuals from the test set. All metabolites were significantly associated with Shannon diversity after adjusting for covariates and multiple testing in the test set (Figure 1). We also tested the predictive value of the MMD score in the test set and we found that it explained 18.68% of the variance in Shannon diversity (Beta (SE) = 1(0.09), *P* = 3.32x10^−26^). We combined the results of the discovery and test set using Fixed Effect Meta-Analysis (Figure 1).

**Figure 2.**
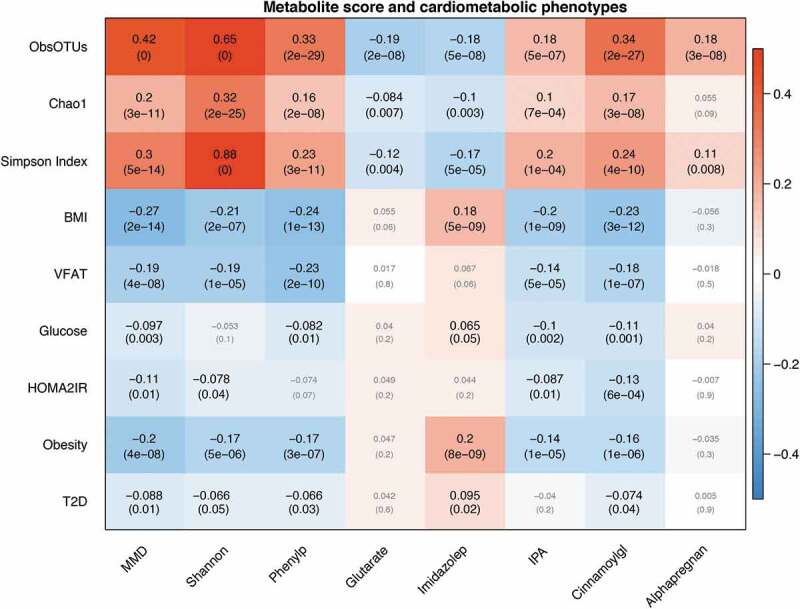
Microbial traits (MMD score, alpha diversity, microbial metabolites), T2D and related traits. Each cell of the matrix contains the correlation coefficient between one microbial trait and a metabolic phenotype score and the corresponding *P* value. The table is color coded by correlation according to the table legend (red for positive and blue for negative correlations). Analyses are adjusted for age and BMI. ObsOTUs = number of observed OTUs, BMI = body mass index, VFAT = visceral fat mass, HOMA2IR = insulin resistance, T2D = type 2 diabetes, MMD = Microbial Metabolites Diversity score, Shannon = Shannon Alpha Diversity Index, Phenylp = 3-phenylpropionate (hydrocinnamate), Imidazolep = imidazole propionate, IPA = indolepropionate, Cinnamoylgl = cinnamoylglycine, Alphapregan = 5alpha-pregnan-3beta,20alpha-diol monosulfate (2).

To further investigate the predictive value of the MMD score, we tested it for association with the other alpha diversity indexes in the overall cohort. As shown in Figure 2 and Table S1, the MMD score was significantly associated with the observed number of OTUs (Beta (SE) = 0.99(0.06), *P* = 2.84x10^−55^; r = 0.42, R2 = 0.19), Simpson Index (0.65 (0.08), P = 5.97x10^−16^; r = 0.3, R2 = 0.08) and Chao1 Index (0.46 (0.07), *P* = 4.21x10^−12^; r = 0.2, R2 = 0.04)

**Figure 3.**
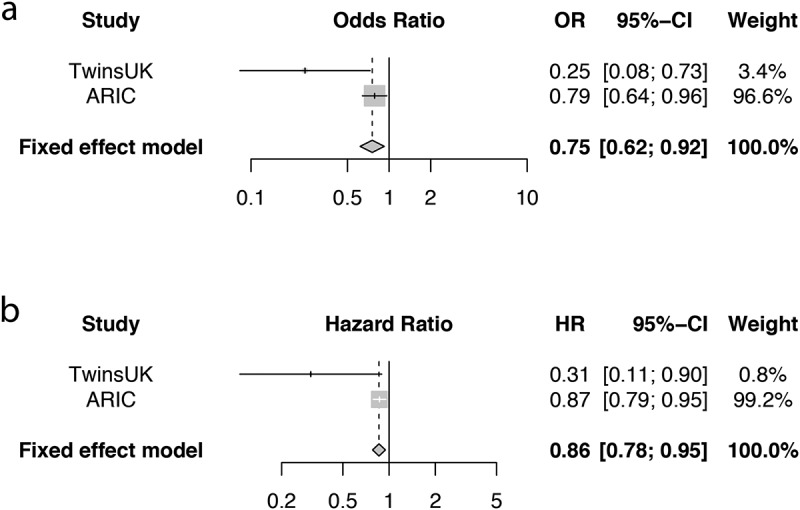
MMD score and risk of (a) prevalent and (b) incident T2D in the TwinsUK (TUK) and ARIC cohorts and results from fixed effect meta-analyses.

We then tested the association between the MMD score and T2D and related phenotypes adjusting for age and BMI. We find that the MMD score was associated with a lower odds of both prevalentT2D (OR[95%CI] = 0.22[0.07;0.70],P = .01) and with lower risk of incident T2D (HR[95%CI] = 0.31[0.11,0.90], *P* = .03). We repeated the adjusting also for baseline fasting glucose and results were consistent. The MMD score was also negatively correlated with BMI, visceral fat mass, fasting glucose and HOMA2-IR, as shown in Figure 2 and in Table S1. We find that the associations between the MMD score and clinical variables were actually stronger than those with actual microbiome diversity (Table S1). To confirm the relevance of the metabolites identified we replicated the metabolite score association with both prevalent and incident T2D in 1522 individuals of European Ancestry from the ARIC cohort. As shown in Figure 3, we find that the MMD score was significantly associated with a lower risk of prevalent and incident T2D both in ARIC (*Prevalent T2D*: OR[95%CI] = 0.79[0.64,0.96], *P* = .02; *Incident T2D*: HR[95%CI] = 0.87[0.79,0.95], *P* = .003) and in the meta-analysis which pooled estimates from TwinsUK and ARIC (*Prevalent T2D*: OR[95%CI] = 0.75[0.62,0.92], Fixed Effect *P* = .006, Han & Eskin^20^ Random-Effect *P* = .006; *Incident T2D*: HR[95%CI] = 0.86[0.78,0.95], *P* = .002, Han & Eskin Random-Effect *P* = .004).

We further replicated the cross-sectional association of the MMD score with BMI (Beta (SE) = −0.59 (0.13), *P* = 5.03x10^−6^)). Considering the gender difference in microbiota and metabolism, we rerun the analysis in ARIC stratifying by gender and we find that the MMD score performance is not always better in females than males though the directionality is always consistent (Table S2).

To test our hypothesis that the association between diversity and T2D is due to the metabolites produced by the microbes we then retested for an association between T2D with the Shannon diversity index after adjusting for metabolites. We found there was no significant association between T2D and alpha diversity when adjusting for the MMD score (OR[95%CI] = 0.81[0.47, 1.38], *P* = .437), while the MMD score is significantly associated to a lower risk of T2D when adjusting for Shannon diversity (OR[95%CI] = 0.26[0.07,0.93], *P* = .038). We therefore conducted formal mediation analysis to determine the indirect effect of the MMD score on the effect between Shannon diversity (high versus low defined as above or below the median, respectively) and T2D. The mediation model, which intends to evaluate the strength of the indirect effects, found that the direct relationship between microbiome factors and T2D was not statistically significant (path coefficient = −.026[−0.06; 0.002]). For indirect effect, the effect of the MMD was statistically significant (path coefficient = −0.24[−0.87; −0.39]). The VAF score for the MMD was 0.28[0.15; 0.94].

## Discussion

In this study, we identified a panel of circulating metabolites reflective of gut microbiome alpha diversity metrics, which was associated with T2D, obesity, adiposity, and other metabolic traits. We found the associations between microbiome diversity and T2D related metabolic traits were stronger with the metabolite surrogate measures than with the actual diversity measures. Our mediation analysis suggests that 28% of the effect the gut microbiome alpha diversity has on T2D is mediated by gut microbiota-associated metabolites. Some of these metabolites are produced by gut bacteria and absorbed through the gut barrier to then reach the bloodstream (3-phenylpropionate, IPA, cinnamoylglycine and imidazole propionate), suggesting that the associations between microbiome diversity and metabolic outcomes are simply reflecting the effect of some of these metabolites. A more diverse microbiome contains a higher number of metabolic pathways and hence is likely to be producing a relatively greater number of metabolites that contribute to lower inflammation and improved metabolic health, such as IPA, cinnamic acid and 3-phenylpropionate. Four of these compounds are the result of microbial amino acid metabolism pathways, one of them is the result of steroid microbial metabolism and one is a phenolic conjugate of a short chain fatty acid.

Importantly, all of the metabolites that we have identified as being strongly predictive of microbiome diversity can be found in human stool samples (see, e.g.,^21^).

A much larger number of metabolites have been shown to be predictive of alpha diversity in a recent study with a smaller sample size^18^ using a LASSO approach. However, less than 25% of those metabolites are of microbial origin, which for our purposes, i.e., to identify metabolites that mediate the effect of the gut microbiome on T2D development, risks confounding causes and effects by including metabolites of purely human origin. In this study, we have focused on identifying metabolites of microbial origin that are individually associated and replicated after adjustment for multiple tests making these much more likely to be implicated in the causal link between gut microbiome diversity and T2D.

### Compounds associated with lower microbiome alpha diversity

Glutarate is produced by the human body as part of the metabolism of tryptophan and lysine. It is produced via lysine degradation in microorganisms^22^ and the microbiome is now known to be an important source of this compound which has been linked to cancer and organic acidurias.^22^ Importantly, glutarate has already been reported to correlate with lower microbiome richness linked to severe obesity.^23^

Imidazole propionate is a microbially produced histidine-derived metabolite.^10^ This compound is present at higher concentrations in the portal and peripheral blood of subjects with type 2 diabetes.^10^ Imidazole propionate can worsen glucose tolerance when administered to mice and impairs insulin signaling at the level of insulin receptor substrate through the activation of p38γ MAPK, which promotes p62 phosphorylation and, subsequently, activation of mTORC1.^10^ The association with obesity, T2D and glucose that we found is therefore not surprising and in fact confirms the results seen in the Dutch cohorts studied for this compound.^10^

### Compounds associated with higher microbiome alpha diversity:

*Indolepropionic acid* (IPA) is a deamination product of tryptophan that can only be produced by gut microbes.^24^ It regulates gastrointestinal barrier function via its interaction with the pregnane X receptor (PXR), which is predictive of the onset of type 2 diabetes,^16^ has been shown to be strongly correlated with higher gut microbiome diversity and its levels in serum are determined primarily by gut microbiome composition.^25^

Among the compounds that intestinal bacteria can metabolize there are sterols, bile acids, and steroid hormones.^26^ The metabolites generated by gut bacteria action on host steroids, when reabsorbed in the intestine back into the circulation, produce biological effects.^26^ The formation of sulfated steroids with a 3 alpha-hydroxy-5 alpha is normally derived from progesterone and account for at least 50% of the metabolism of progesterone in late pregnancy.^27^ Compounds in this class have been implicated in the severity of premenstrual symptoms.^28^ This compound however is also present in human feces^21^ and importantly it has been, along with IPA, reported to be decreased in the plasma of patients with primary dilated cardiomyopathy.^29^ We found that higher serum levels of 5-alpha-pregnan-3beta,20 alpha-diol monosulfate (2) were positively correlated with microbiome diversity and in the context of the metabolite score, with lower BMI, VFAT and risk of T2D. The negative association with primary dilated cardiomyopathy is particularly relevant as that study included both men and women with an average age of 47 y, suggesting that the microbial origin of the metabolite may be the same for both genders. Indeed, Wilmanski and colleagues^18^ reported that serum concentrations of 5-alpha-pregnan-3beta,20 alpha-diol monosulfate (2) were slightly higher in men than in women and that this steroid is associated with microbiome diversity in both males and females.

*3-phenylpropionic acid* is an organic acid that is abundantly found in the products of lactic acid bacterial fermentation^30^ and in honey.^31^ It is an antimicrobial compound with broad-spectrum activity against a wide range of Gram-positive bacteria, some Gram-negative bacteria and fungi.^32^ 3-phenylpropionic acid along with phenylacetic acid is one of the main phenolic metabolites present in human feces. Its levels in feces have been shown to be inversely correlated with fecal levels of propionate and positively correlated with fecal levels of acetate. It appears therefore to be directly related to SCFA metabolism.^33^ Given its correlation with dietary fiber, it is not surprising that it is associated positively with microbiome diversity. The mechanism by which it influences cardiometabolic traits is unknown but we hypothesize that its antimicrobial properties may result in lower production of lipopolysaccharide and that its antioxidant activities may contribute to decreased risk of obesity and insulin resistance.^34^

*Cinnamic acid* is a naturally occurring bioactive compound synthesized in plants by the shikimate pathway, where phenylalanine and tyrosine are two precursor molecules. It is found in plant-based foods such as fruits, vegetables, and whole grains and several potential benefits of cinnamic acid and its derivatives have been demonstrated in *in vitro* and preclinical studies with regards to T2D, although there is no clinical evidence.^35^ Cinnamoylglycine is a glycine conjugate of cinnamic acid and it is known to be produced by gut microbes because it is abundant in the serum of conventional mice but present in minimal concentrations in the serum of germfree mice.^24^ Cinnomoylglycine has substantially greater urinary clearance than creatinine^36^ and hence accumulates in plasma under conditions of diminished renal function. In our data, we found that the association between cinnomoylglycine, diversity and clinical traits was not affected when adjusting for creatinine. Although its functional effects in humans are unknown, urinary excretion levels of cinnamoylglycine have been proposed as markers of colonization resistance against *Clostridium difficile*, i.e., as a marker of a healthy gut microbiome that can inhibit the growth of pathogenic microorganisms.^37^

The current study has several strengths. The study benefits from a big sample size, the use of internal subsets for discovery and retesting, a large independent replication cohort and a large number of metabolites. Both TwinsUK and ARIC have extensive phenotypic data available that allowed us to accurately classify subjects according to T2D status. Finally, the robustness of our result is highlighted by the fact that we confirm many previous findings, and the predictive value of our metabolic score is successfully validated in an independent sample.

Our study has some limitations. Because TwinsUK is a sample representative of the general UK population the number of prevalent and incident cases of T2D in a sample of ~1000 individuals is relatively modest (n = 47 and 23, respectively) hence the large confidence intervals. Moreover, the definitions for T2D are different in the two cohorts. In the TwinsUK subjects, glucose concentration was only measured in the fasting state, potentially underestimating T2D incidence. This and the lack of men in the analysis are study limitations that are at least in part overcome by the inclusion of the ARIC cohort, which includes men and a much larger number of individuals with incident and prevalent T2D. Also, we were not able to test for the association between the MMD score and Shannon diversity and the mediation analysis results in ARIC as data on 16 s gut microbiome data was not available.

In conclusion, in this study, we find that a set of six microbially derived or microbially modified metabolites in serum was predictive of gut microbiome diversity and of T2D risk in two independent populations, and we report that the effect of gut microbiome diversity on risk of T2D is in part mediated by these metabolites. Clinical studies should investigate the pathways identified and determine whether modifying these compounds through diet could help reduce or monitor T2D disease.

## Subject and methods

### Study subjects

We included a random subset of 1018 females from TwinsUK representative of the general middle age female UK population^38^ with concurrent fasting serum metabolomic profiling (592 metabolites) and fecal 16 S sequencing (measuring microbiome composition) along with diabetic and metabolic information. T2D prevalent cases were defined as individuals with fasting glucose≥7 mmol/l or with a physician’s letter confirming diagnosis or use of medication for diabetes. Visceral fat (VF), BMI, obesity and Homa2-IR were measured as previously described.^25^ Incident T2D cases were defined with the same criteria but having developed T2D at follow-up (mean (SD) = 8.16 (1.3) y).

The study was approved by NRES Committee London–Westminster, and all twins provided informed written consent.

The replication cohort consisted of 1522 European American (T2D incident cases 467, T2D prevalent cases 119) from the Atherosclerosis Risk in Communities (ARIC) study with fasting metabolomic profiles.^39^ In the ARIC study, prevalent diabetes at baseline was defined as fasting glucose ≥7.0 mmol/l, non-fasting glucose ≥11.1 mmol/l, self-reported diagnosis of diabetes by a physician or use of medication for diabetes within the previous 2 weeks. The incidence of diabetes was ascertained from visit 1 (1987–1989) until December 31, 2015. Incident diabetes was defined as elevated glucose (fasting glucose ≥7.0 mmol/l or non-fasting glucose ≥11.1 mmol/l) at any of three triennial follow-up visits and visit 5 (2010–2013), self-report of a diabetes diagnosis by a physician at a study visit or annual follow-up telephone interview, or self-report of diabetes medication use during a study visit or annual follow-up telephone interview.^40^ The incidence of diabetes was ascertained from visit 1 until December 31, 2015.

Note, the accumulative T2D incidence per year is 0.4%[0.16%; 1.02%] in the subset of TwinsUK individuals included in this study, 1.1%[0.62%; 1.96%] in the participants from ARIC included here. This compares to 0.67% [0.53%;085%] in US individuals according to the National Diabetes Statistics Report.^41^

### Metabolomics profiling

Metabolomics profiling was conducted using ultra-high-performance liquid chromatography-tandem mass-spectrometry by the metabolomics provider Metabolon Inc. (Morrisville, USA) on fasting serum samples from participants in the TwinsUK study and ARIC, as described previously,^39,42^ for details see Supplementary Text. The metabolomic dataset measured by Metabolon includes 592 known metabolites containing the following broad categories – amino-acids, peptides, carbohydrates, energy intermediates, lipids, nucleotides, cofactors and vitamins, and xenobiotics. These include metabolites of established microbial origin.^43^ We inverse normalized circulating metabolite levels as the metabolite concentrations were not normally distributed. We imputed missing values using the minimum run-day measures.

### Microbiome analysis

Gut microbiome composition was determined by 16 S rRNA gene sequencing carried out as previously described.^44^ Briefly, the V4 region of the 16 S rRNA gene was amplified and sequenced on Illumina MiSeq. Reads were then summarized to operational taxonomic units (OTUs) Quality control was carried out on a per sample basis, discarding paired-ends with an overlap of less than 200nt and removing chimeric sequences using de novo chimera detection in USEARCH.^45^ De novo OTU clustering was then carried across all reads using Sumaclust within QIIME 1.9.0, grouping reads with a 97% identity threshold.^46,47^ OTU counts were converted to log transformed relative abundances, with zero counts handled by the addition of an arbitrary value (10–6). In order to calculate alpha diversity, the complete OTU count table was rarefied to 10,000 sequences per sample 50 times. Alpha diversity metrics were calculated for each sample in each of the rarefied tables and final diversity measures taken as the mean score across all 50. Alpha diversities were quantified as observed OTU counts and Shannon and Simpson diversity indices, commonly used to characterize species diversity in a community. They both account for abundance and evenness of the species present. Alpha diversity indexes were standardized to have mean 0 and SD 1.

### Statistical analysis

Statistical analysis was carried out using Stata version 12 and R version 3.6.0.

In the TwinsUK cohort, we randomly divided study subjects into two independent subsets: a discovery set including 409 individuals (1/3 of the sample) and a test set of 609 individuals (2/3) of the sample. In the discovery set, association analyses between gut microbiome composition and metabolites were performed using random intercept linear regression adjusting for age, body mass index, family relatedness and multiple testing using Bonferroni correction (*P* < 1.68x10^−4^ = 0.1/596 metabolites). We linearly combined the metabolites significantly associated with Shannon Diversity to create a Microbial Metabolites Diversity (MMD) score. We validated the score in 609 females from TwinsUK. Results from the Discovery and Test set were combined using inverse variance fixed effect meta-analysis as implemented in the R package *meta*. We further tested the predictive value of the MMD score by testing its association with T2D and related traits (obesity, BMI, visceral fat, fasting glucose, insulin resistance) and with other measures of diversity in the overall cohort. We replicated the results in 1522 male and female participants of European ancestry from ARIC. In the ARIC study, we applied linear regression to test the association between the MMD score and BMI adjusting for age, sex and center. We next applied logistic and Cox regressions to test the association between the MMD score and prevalent and incident T2D adjusting age, sex, center and BMI. Prevalent T2D cases were excluded from the incident T2D analysis. We did not observe violation on the proportional hazards assumption for MMD score.

Results were combined using inverse variance fixed effect meta-analysis as implemented in the R package *meta* and, since the sex distributions of the two cohorts are different, also using Han & Eskin^20^ random-effect meta-analysis as implemented in the software *metasoft*.

Finally, we employed mediation analysis as implemented in the STATA package MedEff to test the mediation effects of microbial metabolites (indirect effect) on the total effect of microbiome factors (Shannon Index) on T2D adjusting for age and BMI. We constructed a mediation model to quantify both the direct effect of microbiome factors on T2D and the indirect (mediated) effects mentioned above. The model goodness of fit was assessed by standardized path coefficient and effect size (f^2^)^48–50^ yield using the lower and upper bound of the 95% confidence interval. The variance accounted for (VAF) score, which represents the ratio of indirect-to-total effect and determines the proportion of the variance explained by the mediation process, was further used to determine the significance of mediation effect.^51^

## Supplementary Material

Supplemental Material
