# Supplementary material for: Serum metabolites reflecting gut microbiome alpha diversity predict type 2 diabetes
Source: Gut Microbes. 2020 Jun 24;11(6):1632–42. doi: 10.1080/19490976.2020.1778261 (PMC7524143; doi:10.1080/19490976.2020.1778261)
Supplement: Supplemental Material [file KGMI_A_1778261_SM0210.zip › Supplementary information/supplementarytext.docx]

*Metabolomics measurements*

The non-targeted metabolomics analysis was performed at Metabolon (Durham, North Carolina, USA) on a platform consisting of four independent ultra-high-performance liquid chromatography–tandem mass spectrometry (UPLC–MS/MS) instruments, as previously described (1).

Samples were prepared using the automated MicroLab STAR system from Hamilton Company. Several recovery standards were added before the first step in the extraction process for quality control purposes. To remove protein, to dissociate small molecules bound to protein or trapped in the precipitated protein matrix, and to recover chemically diverse metabolites, proteins were precipitated with methanol under vigorous shaking for 2 min (Glen Mills GenoGrinder 2000) followed by centrifugation. The resulting extract was divided into five fractions: two for analysis by two separate reverse-phase (RP)/UPLC–MS/MS methods with positive-ion-mode electrospray ionization (ESI), one for analysis by RP/UPLC–MS/MS with negative-ion-mode ESI, one for analysis by HILIC/UPLC–MS/MS with negative-ion-mode ESI, and one reserved for backup. Samples were placed briefly on a TurboVap (Zymark) to remove the organic solvent. The sample extracts were stored overnight under nitrogen before preparation for analysis.

Several types of controls were analyzed in concert with the experimental samples: a pooled matrix sample generated by taking a small volume of each experimental sample (or, alternatively, a pool of well-characterized human plasma) served as a technical replicate throughout the data set; extracted water samples served as process blanks; and a cocktail of quality control standards that were carefully chosen not to interfere with the measurement of endogenous compounds was spiked into every analyzed sample, allowed instrument performance monitoring and aided chromatographic alignment. Instrument variability was determined by calculating the median relative standard deviation (RSD) for the standards that were added to each sample before injection into the mass spectrometers. Overall process variability was determined by calculating the median RSD for all endogenous metabolites (i.e., non-instrument standards) present in 100% of the pooled-matrix samples. Experimental samples were randomized across the platform run with quality control samples spaced evenly among the injections.

All methods used a Waters ACQUITY ultra-performance liquid chromatographer and a Thermo Scientific Q-Exactive high-resolution mass spectrometer interfaced with a heated electrospray ionization (HESI-II) source and Orbitrap mass analyzer operated at 35,000 mass resolution. The sample extract was dried and then reconstituted in solvents compatible with each of the four methods. Each reconstitution solvent contained a series of standards at fixed concentrations to ensure injection and chromatographic consistency. One aliquot was analyzed using acidic positive-ion conditions, chromatographically optimized for more hydrophilic compounds. In this method, the extract was gradient eluted from a C18 column (Waters UPLC BEH C18–2.1 × 100 mm, 1.7 μm) using water and methanol, containing 0.05% perfluoropentanoic acid (PFPA) and 0.1% formic acid (FA). Another aliquot was also analyzed using acidic positive-ion conditions; however, it was chromatographically optimized for more hydrophobic compounds. In this method, the extract was gradient eluted from the same afore-mentioned C18 column using methanol, acetonitrile, water, 0.05% PFPA and 0.01% FA and was operated at an overall higher organic content. Another aliquot was analyzed using basic negative-ion-optimized conditions on a separate dedicated C18 column. The basic extracts were gradient eluted from the column using methanol and water, however, with 6.5 mM ammonium bicarbonate at pH 8. The fourth aliquot was analyzed via negative ionization following elution from a HILIC column (Waters UPLC BEH Amide 2.1 × 150 mm, 1.7 μm) using a gradient consisting of water and acetonitrile with 10 mM ammonium formate, pH 10.8. The MS analysis alternated between MS and data-dependent MS^n^ scans using dynamic exclusion. The scan range varied slightly between methods but covered 70–1,000 *m*/*z*. Raw data files are archived and extracted as described below.

Raw data were extracted, peak identified and quality control processed using Metabolon's hardware and software. These systems are built on a web service platform using Microsoft's .NET technologies, which run on high-performance application servers and fiber-channel storage arrays in clusters to provide active failover and load balancing. Compounds were identified by comparison to library entries of purified standards or recurrent unknown entities. Metabolon maintains a library based on authenticated standards that contains the retention time/index (RI), mass-to-charge ratio (*m*/*z)* and chromatographic data (including MS/MS spectral data) on all molecules present in the library. Furthermore, biochemical identifications are based on three criteria: retention index within a narrow RI window of the proposed identification, accurate mass match to the library ±10 ppm, and MS/MS forward and reverse scores between the experimental data and authentic standards. MS/MS scores are based on a comparison of the ions present in the experimental spectrum to the ions present in the library spectrum. While there may be similarities between these molecules based on one of these factors, the use of all three data points can distinguish and differentiate biochemicals. More than 3,300 commercially available purified standard compounds have been acquired and registered into LIMS for analysis on all platforms for determination of their analytical characteristics. Additional mass spectral entries have been created for structurally unnamed biochemicals, which have been identified by virtue of their recurrent nature (both chromatographic and mass spectral). These compounds have the potential to be identified by future acquisition of a matching purified standard or by classical structural analysis.

A variety of curation procedures were carried out to ensure that a high-quality data set was made available for statistical analysis and data interpretation. The quality control and curation processes were designed to ensure accurate and consistent identification of true chemical entities and to remove those representing system artifacts, misassignments and background noise. Metabolon data analysts used proprietary visualization and interpretation software to confirm the consistency of peak identification among the various samples. Library matches for each compound were checked for each sample and corrected if necessary.

(1) Long T, Hicks M, Yu HC, Biggs WH, Kirkness EF, Menni C, Zierer J, Small KS, Mangino M, Messier H, Brewerton S, Turpaz Y, Perkins BA, Evans AM, Miller LA, Guo L, Caskey CT, Schork NJ, Garner C, Spector TD, Venter JC, Telenti A: Whole-genome sequencing identifies common-to-rare variants associated with human blood metabolites. Nature genetics 2017;49:568-578
