# Supplementary material for: Serum metabolites reflecting gut microbiome alpha diversity predict type 2 diabetes
Source: Gut Microbes. 2020 Jun 24;11(6):1632–42. doi: 10.1080/19490976.2020.1778261 (PMC7524143; doi:10.1080/19490976.2020.1778261)
Supplement: Supplemental Material [file KGMI_A_1778261_SM0210.zip › Supplementary information/Tables S1S2.docx]

**Table S1. Association between microbial traits, metabolic traits and the MDM score and Shannon Index, adjusting for covariates**

|  | **MMD score** | | **Shannon Index** | |
| --- | --- | --- | --- | --- |
|  | **Beta[95%CI]** | **P** | **Beta[95%CI]** | **P** |
| Observed OTUs | 0.99[0.86;1.11] | 2.84E-55 | 0.66[0.61;0.71] | 7.86E-104 |
| Simpson | 0.65[0.49;0.81] | 5.97E-16 | 0.89[0.82;0.96] | 2.58E-94 |
| Chao1 | 0.46[0.33;0.6] | 4.21E-12 | 0.34[0.28;0.4] | 2.04E-25 |
| BMI | -3.03[-3.78;-2.27] | 3.84E-15 | -0.89[-1.21;-0.56] | 1.49E-07 |
| VFAT mass | -0.28[-0.37;-0.18] | 2.86E-08 | -0.1[-0.15;-0.06] | 1.35E-05 |
| Glucose | -0.14[-0.26;-0.02] | 1.00E-02 | -0.03[-0.07;0.01] | 1.74E-01 |
| HOMA2-IR | -0.14[-0.25;-0.03] | 1.10E-02 | -0.13[-0.24;-0.01] | 2.70E-02 |
|  | **OR[95%CI]** | **P** | **OR[95%CI]** | **P** |
| Obesity | 0.2[0.11;0.36] | 4.50E-08 | 0.57[0.44;0.72] | 5.36E-06 |
| T2D, prevalent | 0.22[0.07;0.69] | 1.00E-02 | 0.64[0.43;0.96] | 3.10E-02 |
|  | **HR[95%CI]** | **P** | **HR[95%CI]** | **P** |
| T2D, incident | 0.31[0.11;0.9] | 3.10E-02 | 0.64[0.43;0.96] | 3.10E-02 |

**Table S2. Association between the MMD score and metabolic traits in the ARIC cohort overall and stratifying by gender. Analyses are adjusted for covariates**

|  | BMI | T2D, prevalent | T2D, incident |
| --- | --- | --- | --- |
|  | *Beta[95%CI]* | *OR[95%CI]* | *HR[95%CI]* |
| All | -0.59[-0.84;-0.34] | 0.75[0.62,0.92] | 0.86[0.78;0.95] |
| Females | -0.45[-0.81;-0.08] | 0.72[0.54;0.96] | 0.93[0.82;1.05] |
| Males | -0.78[-1.1;-0.45] | 0.85[0.64;1.14] | 0.79[0.68;0.91] |
